# Severe Acute Decompensated Heart Failure in a Patient with Cardiac Sarcoidosis

**DOI:** 10.3390/jcm14238462

**Published:** 2025-11-28

**Authors:** Mateusz Lucki, Ewa Straburzyńska-Migaj, Szczepan Cofta, Maciej Lesiak

**Affiliations:** 1 Department of Cardiology, Poznan University of Medical Sciences, 61-701 Poznań, Poland; estraburzynskamigaj@ump.edu.pl (E.S.-M.); mlesiak@ump.edu.pl (M.L.); 2 Department of Pulmonology, Allergology and Pulmonary Oncology, Poznan University of Medical Sciences, 60-569 Poznan, Poland; scofta@ump.edu.pl

**Keywords:** heart failure, acute decompensation, sarcoidosis, late gadolinium enhancement, corticosteroid therapy

## Abstract

**Introduction:** Cardiac sarcoidosis (CS) is a rare but potentially life-threatening manifestation of systemic sarcoidosis, often leading to arrhythmias, conduction abnormalities, or heart failure. Diagnosis is challenging due to nonspecific symptoms and the need for advanced imaging or biopsy. **Case Presentation:** We describe a 49-year-old man admitted with severe decompensated heart failure (NYHA IV). He had a history of complete heart block treated with pacemaker implantation and subsequent CRT-D upgrade. On admission, echocardiography revealed biventricular dysfunction with severe mitral and tricuspid regurgitation. Cardiac MRI demonstrated extensive non-ischemic late gadolinium enhancement. Blood cultures grew methicillin-sensitive *Staphylococcus aureus* (MSSA) and intravenous antibiotics were initiated. Despite diuretics and inotropes, his condition deteriorated. Corticosteroid therapy was started due to high suspicion of sarcoidosis. Endomyocardial biopsy confirmed CS. The patient developed neuropsychiatric complications and, despite urgent listing for heart transplantation, died during hospitalization. **Conclusions:** This case highlights the diagnostic and therapeutic challenges of CS, the limitations of corticosteroid therapy in advanced disease, and the importance of early recognition with advanced imaging modalities.

## 1. Introduction

Sarcoidosis is a multisystem granulomatous disorder of unknown origin, characterized by the formation of non-caseating granulomas in various organs, most commonly affecting the lungs and intrathoracic lymph nodes [1]. The clinical presentation ranges from asymptomatic incidental findings to severe organ dysfunction. Extrapulmonary involvement occurs in approximately 30–40% of patients most frequently involving the heart, eyes, liver and skin [2].

Cardiac sarcoidosis (CS) represents one of the most serious extrapulmonary manifestations, leading to conduction abnormalities, ventricular arrhythmias, or progressive heart failure. Although clinically evident in only 5% of patients with systemic sarcoidosis, cardiac involvement may be present in up to 25% when advanced imaging or autopsy findings are considered [3]. CS is also recognized as one of the leading causes of sudden cardiac death in young and middle-aged adults [4].

The diagnosis of CS remains challenging due to its heterogeneous presentation and the limited sensitivity of conventional diagnostic tools. Multimodality imaging, particularly cardiac magnetic resonance (CMR) with late gadolinium enhancement (LGE) and 18F-fluorodeoxyglucose positron emission tomography (FDG-PET), has markedly improved diagnostic accuracy by allowing detection of both inflammatory and fibrotic myocardial lesions [5,6].

Corticosteroids remain the mainstay of therapy and can improve left ventricular function, when initiated early. though their effect on long-term outcomes and arrhythmic risk remains uncertain [7]. In advanced fibrotic stages. the response to immunosuppressive therapy is often limited, and prognosis remains poor.

Here, we present a case of rapidly progressive cardiac sarcoidosis manifesting as severe acute decompensated heart failure refractory to standard therapy. This case underscores the diagnostic complexity of CS, the limited reversibility of advanced myocardial fibrosis, and the potential complications of corticosteroid therapy, including neuropsychiatric manifestations.

## 2. Methodology

This case report was prepared in accordance with the CARE (CAse REport) guidelines to ensure clarity, completeness, and clinical relevance. Clinical data were obtained retrospectively from the hospital’s electronic medical records (Eskulap Hospital Information System, version **[e.g., 10.3.1]**, CGM Polska, Poznań, Poland) and included demographic information, medical history, physical examination findings, laboratory test results, imaging studies, and therapeutic interventions during hospitalization. All data were fully anonymized prior to analysis. Data extraction and statistical analysis were conducted using PQStat software (version **1.8.4**, PQStat Software, Poznań, Poland). All data were fully anonymized prior to analysis.

As the patient had died, written informed consent for the publication of this case report and any accompanying materials (including clinical images) was obtained from the patient’s legal representative (spouse), who was authorized to access the patient’s medical records in accordance with applicable legal and institutional regulations. The report was prepared using fully anonymized clinical data, without disclosure of any personally identifiable information. The publication complies with the principles of the Declaration of Helsinki and with institutional policies governing the ethical use of data from deceased patients.

## 3. Case Presentation

A 49-year-old man was admitted due to severe decompensated heart failure. Three weeks earlier, the patient had been hospitalized in the Department of Pulmonology for sarcoidosis work-up. At that time, chest computed tomography revealed bilateral hilar and mediastinal lymphadenopathy with multiple subpleural nodules, raising suspicion of systemic sarcoidosis. However, bronchoscopic biopsy was non-diagnostic, and corticosteroid therapy was not initiated. Heart failure had first been diagnosed nine months earlier, when during hospitalization for complete heart block a dual-chamber pacemaker (DDD) was implanted. Coronary angiography performed at that time excluded significant coronary stenoses. At the time of the initial hospitalization for complete heart block, the patient had no clinical signs of heart failure and no extracardiac manifestations suggestive of systemic sarcoidosis. Laboratory testing was unremarkable and echocardiography demonstrated only mildly reduced left ventricular ejection fraction without regional wall-motion abnormalities or morphological “red flags” typically associated with cardiac sarcoidosis. Consequently, the conduction disturbance was attributed to idiopathic disease, and no further sarcoidosis-oriented evaluation was pursued at that stage. This early clinical presentation likely contributed to the diagnostic delay that preceded the subsequent development of overt cardiomyopathy. Due to persistent left ventricular dysfunction (LVEF ≈ 30%) and recurrent heart failure symptoms despite guideline-directed medical therapy, the patient underwent reassessment of his pacing system. Six months prior to the terminal hospitalization, an upgrade to CRT-D was performed. At that time, the ECG demonstrated > 90% right ventricular pacing with a paced QRS duration of 160 ms and a morphology consistent with left bundle branch block. Echocardiography revealed progressive systolic impairment together with typical features of pacing-induced dyssynchrony, including septal flash and apical rocking. Therefore, the decision to proceed with early CRT-D implantation was fully justified and undertaken independently of the yet-unrecognized cardiac sarcoidosis. Family history revealed pacemaker implantation in the patient’s father.

**Figure 1:**
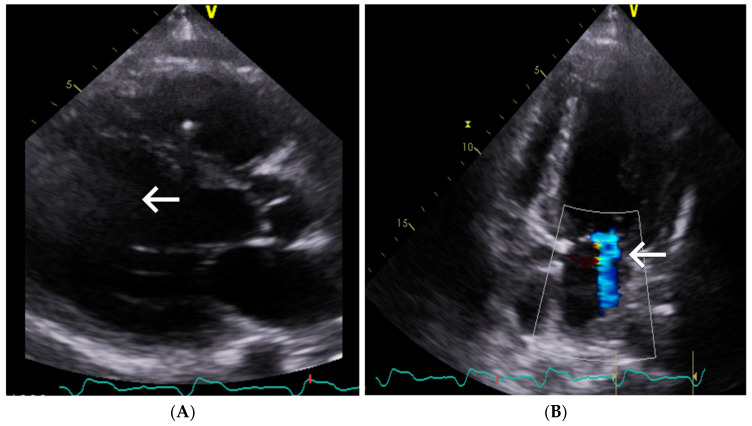
Transthoracic echocardiography. (**A**) Dilated cardiomyopathy with severe left ventricular dysfunction (LVEDD 68 mm, LVEF 30%) and right ventricular enlargement. (**B**) Hemodynamically significant mitral regurgitation visualized on color Doppler. White arrows indicate the key pathological findings in each panel, and color Doppler signals denote the regurgitant jet.

On admission, the patient presented with decompensated heart failure in NYHA functional class IV with exertional dyspnea and orthopnea. Physical examination revealed diminished vesicular breath sounds at the lung bases, ascites. and moderate peripheral edema. Resting electrocardiogram showed sinus rhythm of 60 bpm without ST-segment abnormalities. Transthoracic echocardiography revealed dilated cardiomyopathy (LVEDD 68 mm) with severe left ventricular systolic dysfunction (LVEF 30%), right ventricular enlargement (RV 37 mmTAPSE 10 mm. RV S’ 8 cm/s), and hemodynamically significant mitral regurgitation and moderate tricuspid regurgitation (TRVmax 3 m/s 73 ms) (Figure 1).

**Figure 2:**
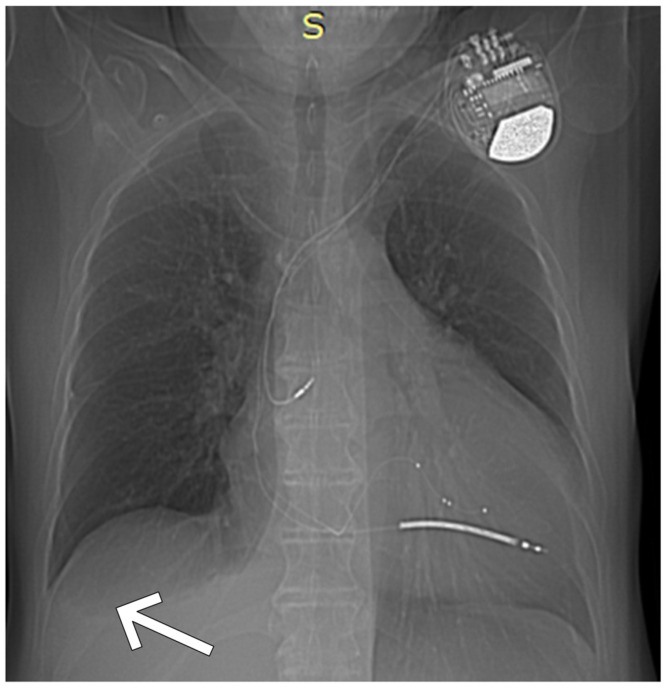
Chest X-ray: enlarged cardiac silhouette and blunting of the right costophrenic angle with pleural effusion. The white arrow indicates the area of effusion.

Chest X-ray demonstrated enlarged cardiac silhouette and blunting of the right costophrenic angle with pleural effusion (Figure 2). Intravenous diuretics and levosimendan were initiated. Due to progressive hypotension, levosimendan was switched to dobutamine. During monitoring, multiple episodes of ventricular tachycardia terminated by ATP and episodes of non-sustained VT were recorded, leading to initiation of amiodarone therapy.

**Figure 3:**
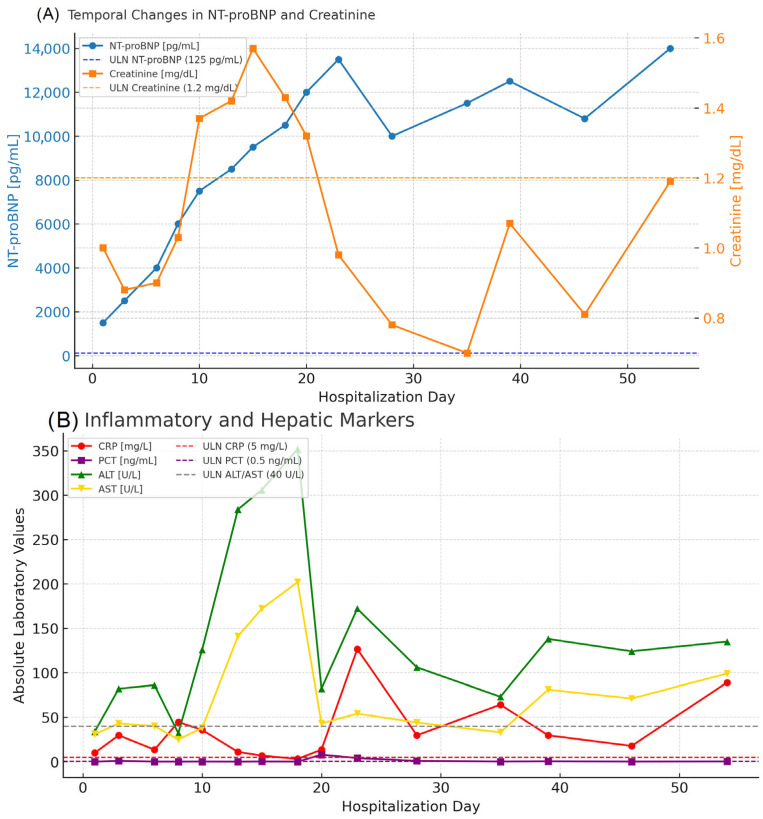
Temporal changes in key laboratory parameters during hospitalization. (**A**) NT-proBNP and creatinine levels showing progressive cardiac and renal dysfunction. (**B**) Inflammatory and hepatic markers (CRP, PCT, ALT, AST) presented as absolute values on a linear scale. Dotted lines of matching color indicate the upper limits of normal (ULN) for each parameter.

Laboratory testing during hospitalization revealed progressive clinical deterioration despite therapy. with marked elevation of NT-proBNP (from 4.774 to 12.891 pg/mL). creatinine (0.72–1.57 mg/dL). CRP (10–136 mg/L). and procalcitonin (0.21–7.59 ng/mL). Blood cultures grew methicillin-sensitive *Staphylococcus aureus* (MSSA), and intravenous cloxacillin was introduced, resulting in gradual reduction in inflammatory markers. Figure 3 presents the temporal changes in key laboratory parameters during hospitalization. NT-proBNP and creatinine levels (Panel A) illustrate progressive cardiac and renal dysfunction, whereas CRP, PCT, ALT, and AST values (Panel B) depict the evolution of systemic inflammation and hepatic involvement. The parameters are shown as absolute values on a linear scale, with dotted lines indicating the upper limits of normal for each marker, providing a clear overview of the patient’s multiorgan deterioration.

**Figure 4:**
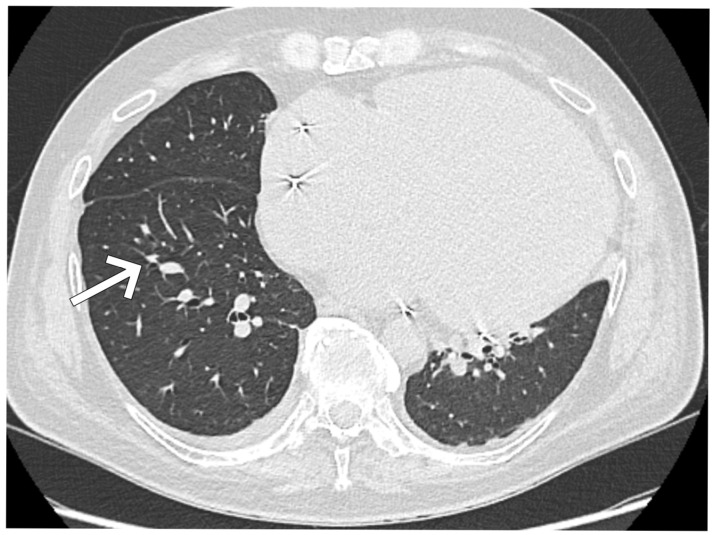
Chest CT (angio-CT) revealing multiple subpleural nodules up to 7 mm and enlarged mediastinal lymph nodes up to 13 mm. The white arrow indicates one of the representative subpleural nodules.

Chest computed tomography revealed multiple subpleural nodules up to 7 mm and enlarged mediastinal lymph nodes to 13 mm (Figure 4).

**Figure 5:**
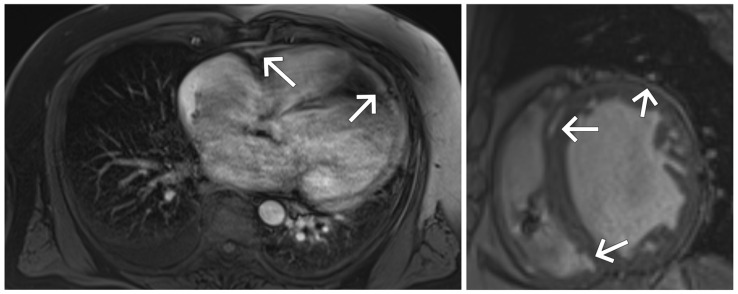
Cardiac magnetic resonance imaging: markedly increased ventricular volumes, and diffuse non-ischemic late gadolinium enhancement of both ventricles shown in long-axis (**left panel**) and short-axis (**right panel**) views. LGE involves subendocardial, mid-wall, and subepicardial layers, consistent with diffuse granulomatous infiltration. White arrows indicate the areas of late gadolinium enhancement.

Contrast-enhanced cardiac magnetic resonance imaging (CMR) was performed. Due to the patient’s resting dyspnea, image acquisition was primarily limited to long-axis planes (two- and four-chamber views). However, a complementary short-axis view was also obtained to better visualize the distribution of late gadolinium enhancement (LGE). The study demonstrated markedly enlarged ventricular volumes and severely reduced global systolic function of both ventricles with diffuse hypokinesis. Additionally, diffuse non-ischemic late gadolinium enhancement was observed, involving subendocardial, mid-wall, and subepicardial layers throughout the left and partially the right ventricular myocardium, consistent with extensive myocardial involvement in sarcoidosis (Figure 5).

**Figure 6:**
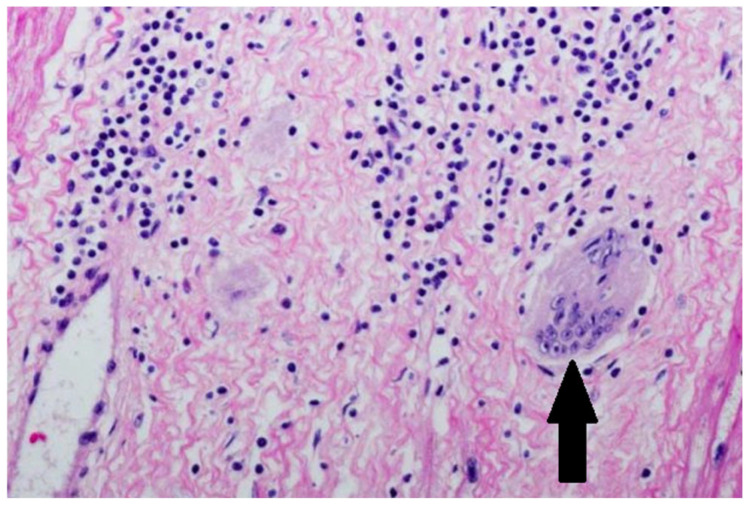
Endomyocardial biopsy (H&E staining. light microscopy. ×400). A well-formed non-caseating granuloma is marked with an arrow, composed of epithelioid histiocytes and multinucleated giant cells within myocardial tissue.

Endomyocardial biopsy ultimately confirmed cardiac sarcoidosis (Figure 6).

The patient was reevaluated by pulmonology. Given the clinical context, sarcoidosis was suspected. On the sixth day of hospitalization, steroid therapy with methylprednisolone was initiated at a dose of 32 mg daily. On the tenth day of hospitalization, the dose was increased to 40 mg daily. On day 10 of corticosteroid treatment, the patient developed neuropsychiatric symptoms including episodes of impaired consciousness, auditory and visual hallucinations, and derealization. Psychiatric consultation led to initiation of risperidone.

Despite supportive care, inotropes, and antibiotic therapy, the patient’s condition progressively worsened with multiorgan dysfunction. The patient was listed for urgent heart transplantation. Table 1 summarizes the clinical evolution, laboratory abnormalities, and key treatment modifications according to the days of hospitalization.

## 4. Discussion

This case illustrates the diagnostic and therapeutic complexity of cardiac sarcoidosis (CS). particularly in patients presenting with acute decompensated heart failure, although the patient had been hospitalized nine months earlier presented with complete heart block. During that ad-mission, the clinical picture was dominated by conduction disturbance without heart failure symptoms, ventricular arrhythmias, or laboratory abnormalities. Echocardiography demonstrated only mildly reduced LVEF, without regional wall motion abnormalities or features typically considered “red flags” for cardiac sarcoidosis (such as basal septal thinning, regional aneurysms, or marked right ventricular dysfunction). At that time, the working diagnosis centered on idiopathic conduction system disease, and no extracardiac manifestations of sarcoidosis were present. This led to pacemaker implantation without further sarcoidosis-oriented evaluation. In hindsight, the absence of systemic features and the nonspecific nature of isolated complete heart block likely contributed to the missed early diagnosis. This reflects real world diagnostic challenges, as early cardiac sarcoidosis may mimic idiopathic conduction disease and often precedes overt cardiomyopathy by months. Therefore, the delay in diagnosis in this case illustrates both the subtlety of early disease and the challenges of recognizing cardiac sarcoidosis before advanced dysfunction develops [2]. In recent years, multimodality imaging—particularly cardiac magnetic resonance (CMR) with late gadolinium enhancement (LGE)—has significantly improved diagnostic accuracy by allowing the detection of both inflammatory and fibrotic myocardial changes [3,4]. In the present case, diffuse LGE involving both ventricles correlated with rapid progression of heart failure and high arrhythmic burden, consistent with previous reports linking extensive fibrosis, especially with right ventricular involvement, to adverse outcomes [5]. Cardiac sarcoidosis can also present with sudden cardiac death as the first and only manifestation, often in patients without prior cardiac symptoms or diagnosis during life. This emphasizes the unpredictable nature of the disease and the importance of early recognition and screening in individuals with systemic sarcoidosis or unexplained conduction disturbances [6,8,9]. What makes this case particularly distinctive is the unique combination of features rarely reported together: biopsy-proven cardiac sarcoidosis with fulminant biventricular involvement, concurrent *Staphylococcus aureus* bacteremia, and the onset of steroid induced neuropsychiatric toxicity. This complex interplay between active myocardial inflammation, systemic infection, and treatment-related adverse effects highlights the real world therapeutic dilemmas clinicians face in managing advanced CS. The clinical course was further complicated by methicillin-sensitive *Staphylococcus aureus* (MSSA) bacteremia, which contributed to systemic inflammation, hemodynamic instability, and progressive multiorgan dysfunction. The marked increase in CRP and procalcitonin levels reflected both infection driven inflammatory response and ongoing myocardial inflammation. Differentiating between sepsis-related inflammation and sarcoid activity remains challenging in such advanced cases and requires cautious interpretation when considering escalation of immunosuppressive therapy [10,11].

The differentiation between infection driven systemic inflammation and sarcoid-related myocardial activity represented a major diagnostic challenge in this case. The patient’s hemodynamic deterioration preceded the confirmation of *Staphylococcus aureus* bacteremia, suggesting that ongoing myocardial inflammation contributed significantly to early clinical decline. The subsequent rise in CRP and procalcitonin likely reflected a dual process—superimposed sepsis and progressive inflammatory cardiomyopathy. Several factors supported this interpretation: the disproportionate increase in NT-proBNP and persistent ventricular dysfunction despite initial antibiotic therapy indicated sustained myocardial injury; and the diffuse. non-ischemic LGE pattern on CMR was consistent with widespread sarcoid-related fibrosis and active inflammation; and the lack of full clinical improvement following appropriate antibiotic therapy implied that infection alone could not account for the worsening course. Therefore. the terminal phase most likely represented a complex interplay between refractory sepsis and advanced cardiac sarcoidosis, where overlapping inflammatory cascades and hemodynamic stress ultimately culminated in multiorgan failure. This dual mechanism is increasingly recognized in the literature as a major contributor to adverse outcomes in patients with end stage cardiac sarcoidosis complicated by secondary infection [6,7,8,9,10,12,13].

Corticosteroids remain the cornerstone of CS management and may improve ventricular function when introduced early, before irreversible fibrosis develops [7,14]. In this case, steroid therapy was initiated after pulmonology consultation and multidisciplinary team discussion, when the combination of progressive clinical deterioration, diffuse non-ischemic LGE on CMR, and extracardiac findings (bilateral hilar and mediastinal lymphadenopathy and subpleural nodules) raised a strong suspicion of CS. An initial moderate oral dose of 32 mg methylprednisolone was intentionally selected instead of high-dose intravenous pulses because of concomitant MSSA bacteremia, markedly elevated procalcitonin levels, and the associated risk of exacerbating infection. The dose was increased to 40 mg on day 10 in response to persistent ventricular dysfunction and the need for stronger immunosuppressive control once early antibiotic therapy had begun to stabilize the bacteremia.

Despite this cautious and guideline aligned therapeutic approach the patient continued to deteriorate, underscoring the limited efficacy of corticosteroids in late stage, fibrotic disease [15]. A challenging aspect of this case was the occurrence of neuropsychiatric complications: hallucinations and derealization, shortly after steroid initiation. Steroid induced psychosis is a well documented adverse effect of corticosteroid therapy [16,17] and in this critically ill patient, it further complicated management and likely contributed to the unfavorable outcome.

Fulminant cases of CS presenting with rapidly progressive biventricular failure are rarely reported, and the coexistence of biopsy confirmed disease, septic complications, and steroid induced neuropsychiatric toxicity is exceptional. Unlike most previously published reports, this patient experienced relentless clinical decline despite early immunosuppression and device therapy, emphasizing the poor reversibility of advanced fibrotic involvement and the importance of timely diagnosis [18,19,20]. Extensive myocardial scarring demonstrated by diffuse LGE remains one of the strongest predictors of mortality and arrhythmic events in CS [21]. Once irreversible fibrosis has developed, immunosuppressive therapy offers limited benefit, and heart transplantation may represent the only definitive treatment option [22,23].

This case provides several important and distinctive diagnostic and therapeutic insights that expand the current understanding of advanced cardiac sarcoidosis. It illustrates an exceptionally rare constellation of fulminant, biopsy-proven disease with extensive biventricular involvement occurring concurrently with MSSA bacteremia—a combination scarcely described in the literature. It also highlights a diagnostic pitfall in which isolated complete heart block, in the absence of systemic manifestations, delayed consideration of sarcoidosis and underscores the importance of early evaluation for infiltrative or inflammatory etiologies in patients presenting with unexplained conduction abnormalities. Furthermore, this case vividly demonstrates the practical challenges of balancing the need for urgent immunosuppression with infection control, including the occurrence of neuropsychiatric complications of corticosteroid therapy in a critically ill patient. Despite device therapy, early CRT-D implantation, and initiation of corticosteroids, the relentless progression toward end-stage heart failure reflects the limited reversibility of diffuse, LGE-positive myocardial fibrosis. Taken together, these features offer valuable teaching points regarding early recognition, risk stratification, and multidisciplinary management of advanced cardiac sarcoidosis.

In summary, this case underscores the need for heightened clinical vigilance in patients with unexplained cardiomyopathy or conduction disturbances and highlights the crucial role of advanced multimodality imaging in early detection and longitudinal disease monitoring. It also emphasizes the importance of individualized therapeutic decision-making and close multidisciplinary collaboration—involving cardiology, pulmonology, radiology, and intensive care specialists—particularly in complex scenarios where active myocardial inflammation coexists with systemic infection. Such coordinated care is essential for optimizing management and improving outcomes in cardiac sarcoidosis. A limitation of this report is the lack of post-mortem confirmation of the diagnosis, as an autopsy was not performed because the patient’s family did not provide consent. Additionally, serum angiotensin-converting enzyme (ACE) and soluble interleukin-2 receptor (sIL-2R) were not measured in this patient. This was due to the rapidly progressive clinical course, acute hemodynamic instability, and concurrent methicillin-sensitive Staphylococcus aureus bacteremia, which necessitated prioritizing urgent therapeutic interventions. In addition, these tests are not available in our hospital laboratory and typically require referral to specialized centers, making their determination impractical in the acute setting. The absence of these biomarkers limited the biochemical assessment of systemic granulomatous activity; however, multimodality imaging and subsequent histopathological confirmation ultimately established the diagnosis. It is important to note that, although ACE and sIL-2R are included in the Japanese Circulation Society (JCS) 2016 guidelines as supportive criteria for the clinical diagnosis of cardiac sarcoidosis, their diagnostic performance is limited [24]. Several studies have demonstrated wide variability in sensitivity and specificity of ACE (sensitivity ranging from 22% to 80%) [25] and only moderate diagnostic value of sIL-2R, even in systemic sarcoidosis [26]. Furthermore, evidence indicates that these biomarkers perform poorly in isolated cardiac involvement and cannot reliably confirm or exclude cardiac sarcoidosis. Therefore, the lack of ACE and sIL-2R measurements in this case did not alter the diagnostic approach, as current guidelines emphasize that advanced cardiac imaging and endomyocardial biopsy remain decisive for definitive diagnosis, particularly in patients with rapidly progressive myocardial dysfunction.

Furthermore, corticosteroid therapy was initiated at a moderate oral dose rather than as high-dose intravenous pulses. This decision was influenced by the concurrent *Staphylococcus aureus* bacteremia and the early onset of neuropsychiatric symptoms, both of which significantly increased the risk associated with aggressive immunosuppression. While this cautious approach may have limited the anti-inflammatory efficacy of treatment, it reflected a necessary balance between infection control and the prevention of further neuropsychiatric deterioration in a critically ill patient.

## 5. Conclusions

Cardiac sarcoidosis should be considered in patients with unexplained cardiomyopathy, conduction disturbance,. or ventricular arrhythmias, particularly in the presence of extracardiac sarcoidosis. Early use of advanced imaging, including CMR and PET, is essential to establish the diagnosis and guide immunosuppressive therapy. Once extensive fibrosis has developed, treatment options are limited, and the prognosis remains poor. Our case emphasizes the need for heightened clinical suspicion and earlier intervention to improve outcomes.

## Figures and Tables

**Table 1:** Summary of clinical evolution and treatment during hospitalization.

Day of Hospitalization	Clinical Symptoms and Events	Cardiac Rhythm Disturbances	Treatment/Procedures	Key Laboratory Abnormalities	Remarks
1	Severe dyspnea. orthopnea. peripheral edema	Sinus rhythm	IV diuretics. levosimendan	↑ NT-proBNP (4.775 pg/mL). mild ↑ CRP (10 mg/L)	Admission. start HF treatment
3	Persistent dyspnea		Dobutamine started due to hypotension	Creatinine ↑ to 0.9 mg/dL NT-proBNP (1.647 pg/mL)	Hemodynamic instability
6	Ascites. edema. worsening fatigue	Nonsustained VT	Furosemide + Opacorden	WBC ↑ (7.8 × 10^9^/L)-normal. mild ↑ CRP (13.6 mg/L)	Planned myocardial biopsy
8	Dyspnea improving slightly	Occasional VT	Metypred 32 mg initiated	Creatinine ↑ to 1.0 mg/dL. NT-proBNP 5.708 pg/mL	Start of corticosteroids
10	Partial clinical improvement		Steroids ↑ to 40 mg	↑ CRP (136 mg/L). ↑ PCT (7.59 ng/mL). ↑ creatinine (1.37 mg/dL)	MSSA bacteremia diagnosed
13	Fluctuating dyspnea		IV cloxacillin started	CRP (11.1 mg/L) decreasing. WBC ↑ (8.5 × 10^9^/L) normal	After antibiotics initiation
15	Hallucinations. derealization	Ventricular arrhythmia episodes	Risperidone added	Creatinine stable (1.4 mg/dL). ↑ AST/ALT (306/172 U/L)	Steroid neuropsychiatric complications
18	Persistent fatigue		Continuation of therapy	↑ NT-proBNP (11.173 pg/mL → upward trend)	Progressive HF
20	Dyspnea. hypotension		Inotropes continued	NT-proBNP > 10.000 pg/mL	Advanced HF
23	Worsening congestion		Furosemide intensified	WBC ↑ (29.2 × 10^9^/L). CRP 1.435 mg/L. PCT 0.98 ng/mL	Sepsis progression
28	Partial stabilization		Ongoing antibiotics	NT-proBNP 12.819 pg/mL. creatinine 0.78 mg/dL	Transient improvement
35	Increasing fatigue. liver congestion		Supportive therapy	↑ ALT 64 U/L. ↑ AST 73 U/L. ↑ bilirubin (1.8 mg/dL)	Liver dysfunction
39	Dyspnea. edema			NT-proBNP 18.780 pg/mL. ↑ creatinine 1.07 mg/dL	Persistent HF
48	General deterioration			↑ NT-proBNP 22.870 pg/mL. ↑ CRP 228.7 mg/L. ↑ GGT 124 U/L	Preterminal state
54	Cardiogenic shock and multiorgan failure.		Urgent HTx listing	NT-proBNP 11.005 pg/mL. creatinine 4.13 mg/dL. ↓ RBC (3.1 × 10^12^/L). ↓ Hb (9.8 g/dL)	Death during hospitalization

↑ indicates increased/elevated values; ↓ indicates decreased/reduced values; → indicates stable values with no significant change.

## Data Availability

The original contributions presented in this study are included in the article. Further inquiries can be directed to the corresponding authors.

## Abbreviations

The following abbreviations are used in this manuscript:

CS	Cardiac sarcoidosis
HF	Heart failure
ADHF	Acute decompensated heart failure
NYHA	New York Heart Association (functional class)
ECG	Electrocardiogram
CRT-D	Cardiac resynchronization therapy with defibrillator
ICD	Implantable cardioverter-defibrillator
LV	Left ventricle/ventricular
RV	Right ventricle/ventricular
LVEDD	Left ventricular end-diastolic diameter
LVEF	Left ventricular ejection fraction
TAPSE	Tricuspid annular plane systolic excursion
TRVmax	Tricuspid regurgitant jet velocity (maximum)
ACT	Acceleration time (pulmonary flow)
CMR	Cardiac magnetic resonance
LGE	Late gadolinium enhancement
PET	Positron emission tomography
CRP	C-reactive protein
NT-proBNP	N-terminal pro-B-type natriuretic peptide
MSSA	Methicillin-sensitive *Staphylococcus aureus*
ATP	Antitachycardia pacing
